# KRAS Mutations Are Associated with Shortened Survival in Patients with Epithelioid Malignant Pleural Mesothelioma

**DOI:** 10.3390/cancers15072072

**Published:** 2023-03-30

**Authors:** Margherita Vannucchi, Veronica Pennati, Clelia Mencaroni, Chiara Defraia, Ledi Bardhi, Francesca Castiglione, Cristiana Bellan, Camilla Eva Comin

**Affiliations:** 1 Section of Pathology, Department of Medical Biotechnology, University of Siena, 53100 Siena, Italy; 2 Division of Pathological anatomy, Department of Medical and Surgical Critical Care, University of Florence, 50121 Florence, Italy; 3 Department of Experimental and Clinical Medicine, Section of Surgery, Histopathology and Molecular Pathology, University of Florence, 50121 Florence, Italy

**Keywords:** pleural mesothelioma, *KDR* gene, *KRAS* mutation, epithelioid mesothelioma, MPM prognosis

## Abstract

**Simple Summary:**

Malignant pleural mesothelioma (MPM) is an aggressive tumor with implacable prognosis. It commonly harbors loss-of-function mutations in *BAP1, NF2, CDKN2A* and *TP53,* although their pathogenetic role is still uncertain since these genetic alterations, if isolated, do not cause MPM in mice models. The aim of our study was to describe the mutational landscape in a cohort of 29 patients with MPM and, eventually, correlate mutation profile with MPM phenotypic traits and prognosis. We report herein a correlation between the presence of *KRAS* mutations and prognosis of the epithelioid MPM and, moreover, the number of mutations correlated with shortened survival. Conversely, improved overall survival was associated with the presence of SNP p.V297I of the *KDR* gene. With the limitation of a relatively small series, these results contribute to the characterization of the mutation profile of MPM and its impact on prognosis.

**Abstract:**

Malignant pleural mesothelioma (MPM) is an aggressive malignancy of the pleural surface that includes three major histologic subtypes, epitheliod, sarcomatoid and biphasic. Epithelioid mesothelioma is usually associated with better prognosis. The genetic mechanisms driving MPM, the possible target mutations and the correlation with overall survival remain largely unsettled. We performed target exome sequencing in 29 cases of MPM aimed at identifying somatic mutations and, eventually, their correlation with phenotypic traits and prognostic significance. We found that *KRAS* mutations, occurring in 13.7% of cases, were associated with shortened median survival (7.6 versus 32.6 months in *KRAS* wild-type; *p* = 0.005), as it was the occurrence of any ≥3 mutations (7.6 versus 37.6 months; *p* = 0.049). Conversely, the presence of *KDR* single nucleotide polymorphism p.V297I (rs2305948) resulted in a favorable variable for survival (NR versus 23.4 months; *p* = 0.026). With the intrinsic limitations of a small number of cases and patient heterogeneity, results of this study contribute to the characterization of the mutation profile of MPM and the impact of selected somatic mutations, and possibly KDR polymorphism, on prognosis.

## 1. Introduction

Malignant pleural mesothelioma (MPM) is a relatively rare and aggressive neoplasm arising from mesothelial cells that line the serous cavities of the body. MPM is the most frequent among malignant mesotheliomas, accounting for 80–95% of cases, followed by peritoneal mesothelioma (5–20%). Other rare cases of mesothelioma originating from pericardium and tunica vaginalis of the testis have also been described [1,2]. When it involves the pleura, symptoms include breathlessness due to pleural effusion, cough, and chest pain associated with malaise and weight loss.

Exposure to asbestos fibers is the most important, well-documented risk factor implicated in the development of MPM, which represents the primum movens in about 70% of patients. In a minority of cases, previous radiotherapy, Simian virus 40, and exposure to other types of fibers such as erionite have been implicated as well. The development of MPM is an asbestos dose-independent process [3] with a long period of latency (10–30 years) between exposure and the cancer’s onset. For this reason, although many countries in the world have banned the use of asbestos, the incidence of MPM is expected to increase until 2025 [4].

According to the 5th edition (2021) of the WHO classification of thoracic tumors, MPM [5] is histologically classified as epithelioid, sarcomatoid and biphasic. Sarcomatoid and biphasic mesothelioma is characterized by a worse overall survival when compared with the epithelioid one [6], with a median OS of 14.4 months for the epithelioid histotype, 9.5 months for the biphasic and 5.3 months for sarcomatoid type [7].

Patients with early stage (stage I-IIIA) disease are treated with combination therapies of surgery, chemotherapy, and radiation, but the choice of surgical approach and the timing of each treatment modality are still controversial. Cisplatin and Permetrexed are currently used to treat patients with advanced stages or those who cannot undergo surgery. Due to marginal survival benefits associated with these treatments, new immunotherapy options appear promising [8,9].

The rationale of using immunotherapy lies in the crucial role of chronic inflammation in MPM development and outgrowth, as supported by results of the CheckMate 743 trial, where first-line therapy with Nivolumab plus Ipilimumab in unresectable MPM outperformed standard chemotherapy [10].

Accordingly, in the phase 3 CONFIRM trial, Nivolumab outperformed placebo in patients who had progressed following platinum-based chemotherapy [11,12].

To date, the lack of an effective cure for MPM empathizes the need to improve our understanding of molecular alterations in the context of clinical, histologic and molecular MPM heterogeneity for the purpose of personalizing and tailoring diagnostic and therapeutic regimens.

MPM is mostly a sporadic neoplasm, and according to the COSMIC database and available information, its genetic landscape has only partially been characterized, although some distinct mechanisms of carcinogenesis have been highlighted. The genomic profile of MPM is characterized by high inter-patient and intra-tumor heterogeneity, a low somatic mutational burden with no overt differences among histologic subtypes [13].

The most common point mutations and somatic copy number alterations affect *RNA* helicase family (*DDX3X, DDX51*), the histone methyltransferase genes (*SETDB1, SETD2*) [14,15,16,17] and tumor suppressor genes (*BAP1, CDKN2A, NF2, TP53, TCSC1, LATS1, LATS2, BLM*, etc.), leading to a loss of function. 

Among them, *BAP1* and *BLM* abnormalities may represent germline gene inactivation in a small subset of patients, increasing predisposition to tumorigenesis.

On the contrary, genetic alterations involving a gain of function of different oncogenes (*PI3K3CA, EGFR, BRAF, HRAS, NRAS, KRAS*, etc.) are less frequently reported. 

Furthermore, occurrence of copy number alterations (CNAs) is well documented and can be found in 70% of cases as chromosomal losses (involving chromosome 1p, 3p, 6q and 9p21 at the locus of the *CDKN2A* and *MTAP* genes) and as chromosomal gains in 30%, especially for 17q involving the cancer-related genes *MAP3K3, SMARD2, ERN1* [18,19,20,21].

In the last 10 years, several studies have addressed the molecular structure of diffuse MPM. However, the genetic mechanisms driving MPM remain largely unsettled, and clearly identifiable predictive and prognostic biomarkers are still lacking.

In order to contribute to the knowledge of somatic mutation landscape and, eventually, to its association with phenotypic traits and prognosis, we performed target exome sequencing in 29 cases of epithelioid MPM. 

## 2. Materials and Methods

### 2.1. Patients and Tissue Samples

We retrospectively analyzed 29 out of 54 MPM tissue samples, both small pleural thoracoscopic biopsies and tissue from pleurectomy/decortication procedure, for which enough material for genomic evaluation was available. Samples were collected from first-diagnosed patients referred to the University-Hospital of Florence, Careggi, between January 2013 and December 2017. The specimens were stored and then collected from the routine archive at our institute.

Hematoxylin/eosin-stained (H&E) slides were reviewed according to the most recent edition of pleura tumor classification [1] by two pathologists (C.E.C and M.V.) who also visually selected the area with at least 50% of tumor cells for genomic DNA extraction. Clinical–pathological characteristics, including age, sex, exposure to asbestos, survival and histologic subtype, were retrieved from chart records. Detailed information concerning treatment was available for a minority of cases; therefore, it was not included in the analysis. All cases were anonymized with a number code.

### 2.2. Genomic DNA Extraction

DNA was obtained from tissue after manual dissection of selected areas containing at least 50% of neoplastic cells.

Genomic DNA (gDNA) was extracted from formalin-fixed-paraffin-embedded samples using MagCore gDNA FFPE one-step kit (Diatech-labline pharmacogenetics, Jesi, Italy). With Myriapod NGS-L T56G Oncopanel Kit (Diatech-labline pharmacogenetics, Jesi, Italy), gDNA fragmentation was evaluated through q-PCR (Rotor-gene, Quiagen, Hilden, Germany) and then amplified with multiplex PCR.

After purification and indexing phase with barcodes, the libraries were quantified using Qubit dsDNA HS Assay kit (Invitrogen, Thermo Fisher Scientific, Mississauga, Canada) and diluted in nuclease-free water to a final concentration of 100 nM.

Emulsion PCR was performed on Ion One Touch 2 (Life technology, Grand Island, NE, USA) while Ion Touch ES Ion Torrent Dynabeats MyOne Streptavidin C1 Beads (Invitrogen, Thermo Fisher Scientific, Mississauga, ON, Canada) were added to the pool.

### 2.3. Next-Generation Sequencing and Variant Calling

DNA sequencing was performed using Ion S5 System Ion Torrent S5^TM^ platform (Invitrogen, Thermo Fisher Scientific, Mississauga, ON, Canada). Tumor samples were tested with a commercial library kit (Myriapod NGS-LT Onco panel 56G, Diatech Pharmacogenetics, Jesi, Italy) targeting over 500 hotspot mutations in 48 genes.

The variant calling was obtained according to Myriapod NGS Data Analysis Software (Diatech Pharmacogenetics, Jesi, Italy), and the reference genome was GRCh37. Only samples sequenced with a quality score (PHRED score) of at least AQ20 were considered, and the coverage was set at a minimum average depth of 100 reads for each amplicon.

Genetic annotation was performed using COSMIC, LOVD, PUBMED and Clinvar for already known genetic variants, while for single-nucleotide polymorphism (SNP), we used dbSNP and 1000 Genome.

Pathogenicity prediction was studied for the variants not yet described using Web ANNOVAR (Variant Annotation) and Provean (http://provean.jcvi.org/index.php (accessed on 27 March 2023)), SIFT (http://sift.jcvi.org/ (accessed on 27 March 2023)) and Pholyfen (http://genetics.bwh.harvard.edu/pph2/ (accessed on 27 March 2023)) prediction software.

### 2.4. Statistical Analysis

Overall survival (OS) was defined as the time from the date of diagnosis to death or last follow-up using Kaplan–Meier method; hazard ratio (HR) was calculated with Cox proportional regression model, and the statistical differences between clinical–pathological variables were obtained using Chi-squared test. A *p* value < 0.05 was considered statistically significant. Statistical analysis was elaborated using SPSS v.25 (IBM, Armonk, NY, USA). 

## 3. Results

### 3.1. Patients Characteristics and Clinical Outcomes

The current series included 25 male (86.2.%) and 4 female (13.7%) subjects. Median age at diagnosis was 74.2 years (range: 63–84). At the time of the study database lock, 21 patients had died (72.4%), whereas 8 were still alive (27.5%) with a median survival of 30.8 months (range: 1.1–248.8). Clinical information about asbestos exposure was available for 25 out of 29 patients (86.2%). Based on the National Mesothelioma Registry criteria (ReNaM, art. 36, D.Lgs 277/91), previous exposure to asbestos fibers was assessed as “certain” in 18 patients (62.0%), “possible” in 3 (10.3%), “probable” in 1 (3.4%) and “unknown” for 7 cases (24.1%). Patient’s comorbidities were not included in the population characteristics since they were not relevant for the study. 

According to histopathology, all 29 MPM cases were epithelioid subtype. Moreover, the slides were re-evaluated, and based on the pathological grading proposed by the WHO of Thoracic Tumors 5th edition, they were subclassified as grade 1 (58.6% *n* = 17), grade 2 (31% *n* = 9) and grade 3 (10.3% *n* = 3). All the patients were in pT1 pathological stage (sec AJCC, 8th ed.). In detail, in 19 cases (65%), MPM was limited to the parietal pleura, 27.5% (*n* = 8) involved parietal and visceral pleura, and in 2 patients (6.8%) the parietal and the diaphragmatic pleura. All the clinical–pathological characteristics are summarized in Table 1.

### 3.2. Somatic Mutations Landscape in MM

NGS analysis revealed a total of 36 somatic mutations. At least one somatic variant was discovered in 23/29 samples (79.3%)**.** In 11 patients (37.9%), NGS sequencing demonstrated the presence of a single mutation, while 2 mutations and 3 or more mutations were identified in 5 (18.5%) and 7 patients (25.9%), respectively. 

*EGFR*, *KDR*, *TP53* and *KRAS* were the most frequently mutated genes, being discovered in about the 62% of the population, corresponding to 18 of 29 patients.

Additional somatic mutations (detected with a frequency of 2–10% of cases) were observed in *CTNNB1*, *RB1*, *STK11*, *FLT3*, *BRAF*, *AKT1*, *HNF1A*, *APC*, *MET*, *SMAD4*, *PTEN*, *KIT*, *MA2K1*, *TSC2*, *ATM*, *CDH1*, *FGFR3*. All the mutations and related variant allele frequencies (VAF) are summarized in Table 2.

The somatic variant with the highest allelic frequency (40.2%) was p.N371K in the *MET* gene, followed by the p.G13A mutation in exon 2 of *KRAS* (VAF =33.5%) and the p.R175H variant in exon 5 of *TP53* (VAF = 32.6%). 

**Figure 1:**
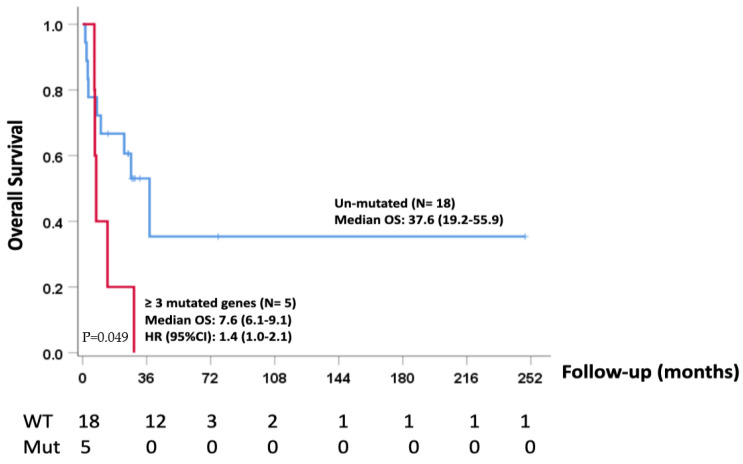
MPM with ≥3 mutations demonstrating a shorter overall survival.

Kaplan–Meier analysis of survival demonstrated that median overall survival was significantly shorter in patients with three or more mutations (median 7.6; range 6–9.1) (*p* = 0.004) compared to patients without mutations (median: 37.6; range: 19.2–56.0) (Figure 1). No significant differences in terms of overall survival were found between cases with more than three mutations when compared with cases carrying one or two alterations.

### 3.3. Identification of Novel EGFR Mutations

We found eight EGFR activating point mutations (22.2%, *n* = 36) in 5/29 patients (17.2%): six of these were considered rare variants (p.V774M, p.T751I and p.S752Y in exon 19; p.G719S and p.A702D in exon 18; p.E66K in exon 21) that are usually described in non-small cell lung cancer. Moreover, we found two SNVs, p.S752C in exon 19 and p.P694T in exon 18, not previously reported, that were considered as functionally relevant by in silico analysis (ANNOVAR Variant Annotation). Moreover, the somatic variant p.G719S was described in two patients.

Two patients presented a double *EGFR* mutation: p.A702D associated with p.S752C in one patient and p.T751I plus p.E866K in the second one.

Statistical analysis did not reveal any correlation between EGFR genetic alterations and either clinical characteristics or survival [22].

### 3.4. TP53 Mutations

We detected six *TP53* somatic mutations (16.6%, *n* = 36) in 4/29 patients (13.7%).

One patient showed two coexisting mutations, the first located in exon 5 (p.P152S) while the second, was a stop variant (p.R213*) in exon 6. All *TP53* mutations were located in exons 5 and 6 (between codons 125 and 300), in the hotspot sites that encode for specific DNA binding domains [23,24,25,26].

In this cohort, mutations in *TP53* did not show any correlation with overall survival or clinical characteristics.

### 3.5. KRAS Mutations

A *KRAS* mutation was found in 13.7% of the patients in the cohort.

Genetic analysis revealed five point mutations (13.8%, *n* = 36) located in exon 2 codon 13 (p.G13A and p.S17N) and in exon 3 codons 48 and 57 (p.G48R, p.G48E and p.D57N), at the sites that encode for the DNA binding domain.

**Figure 2:**
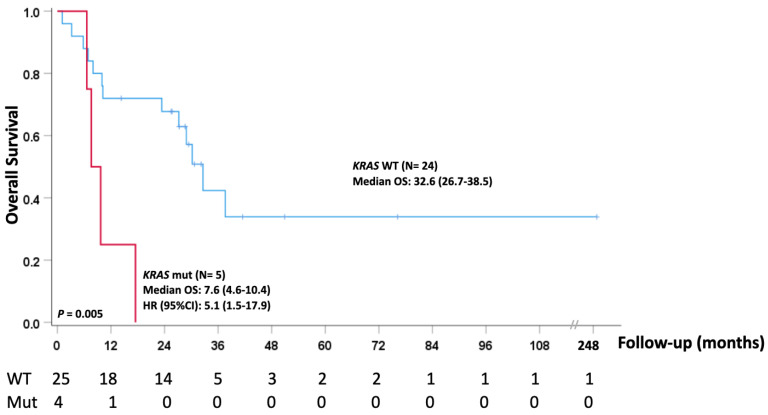
Epithelioid MPM-*KRAS* mutated vs. epithelioid MPM-KRAS wild type.

Kaplan–Meier analysis revealed a negative impact of *KRAS* mutations on survival. In detail, the median overall survival was 7.6 months (range: 4.6–10.4) for patients carrying the *KRAS* genetic alteration, while OS was significantly longer in *KRAS* wild-type cases: 32.6 ((range: 26.7–38.5) with a hazard ratio (HR) of (95%CI): 5.1 (1.5–17.9) (*p* = 0.005)) (Figure 2). Notably, all four *KRAS*-mutated patients reported a certain previous occupational asbestos exposure. However, we found no concurrent *KRAS* and TP53 mutations and no correlation between *KRAS* somatic alteration and clinical features.

### 3.6. Single Nucleotide Polymorphism (SNP)

Next-generation sequence analysis discovered a series of SNPs in the *TP53, SMAD4*, *KDR* and *STK11* genes. We focused our attention on the single-nucleotide polymorphism p.V297I of the *KDR* gene found 5 of 29 cases (17.2%).

Kaplan–Meier analysis showed a longer overall survival (*p* = 0.026) in patients harboring this SNP with a median overall survival not reached (NR) as compared to patients without this particular *KDR* SNV, who showed a median overall survival of 23.4 months (0.3–48.9, HR (95%CI): 0.32 (0.02–0.95)).

**Figure 3:**
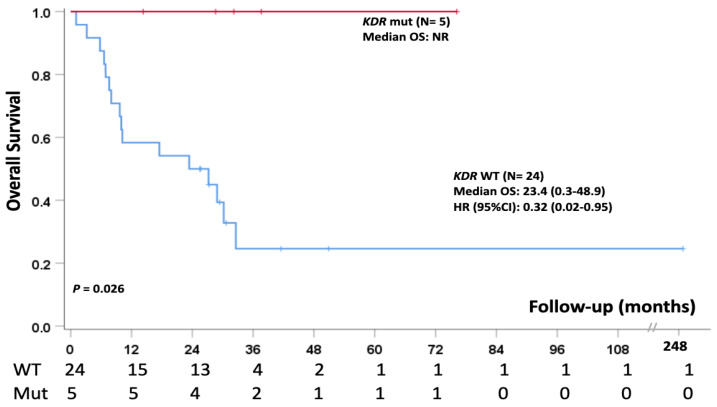
SNP p.V297I of the *KDR* gene confers a significantly better prognosis in MPM patients.

This evidence suggests that SNP p.V297I in the *KDR* gene might be considered a favorable prognostic factor in epithelioid MPM patients (Figure 3).

## 4. Discussion

Pleural mesothelioma is an aggressive tumor with implacable prognosis often associated with asbestos exposure, and to date, there is no real knowledge about the pathogenetic mechanisms of this neoplasm. Moreover, because of its rarity, the number of patients tested in most studies is small [27].

The emerging use of next-generation sequencing, however, has highlighted an increasing number of mutated genes that may have a role in MPM biology.

Mesothelioma usually harbors a low mutation burden (TMB) and low genetic complexity with an overall rate of <2 non-synonymous mutations per megabase [16,28], although cases carrying several gene mutations and/or co-mutations have been reported. 

MPM is preferentially associated with alterations in gene involved in the cell cycle, Hedgehog and Hippo pathway, and in the DNA repair mechanism. Furthermore, the signaling pathways most frequently affected are the PI3K/AkT/mTOR and MAPK pathways [29,30].

Another important mechanism impaired in MPM is the epigenetic regulation, particularly due to the hypermethylation of three genes: TMEM30B, KAZALD1 and MAPK13.

In a recent study [29], genomic analysis of more than 1000 pleural mesothelioma samples showed that the most frequent molecular alteration was a loss of function (LOH) of specific tumor suppressor genes such as CDKN2A, CDKN2B, BAP1 and NF2 (15.20% of the total cases each), which is in line with all previous studies.

Notably, additional mutations in targetable genes such as KRAS, EGFR, PDGFRA/B and FGFR3 were detected, although with a frequency of <5% in their population.

The authors of this study, based on the presence, absence or co-occurrence of CDKN2A/B and BAP1 mutations, identified four mesothelioma subgroups with different prognostic impact. 

Moreover, a novel molecular MPM subtype has been described. This new entity showed a distinctive clinical phenotype, being frequent in female patients with younger age at diagnosis and being recurrent in TP53 and SETDB1 mutations [20].

Gain-of-function mutations in MPM patients are infrequent. Copy number gains and hotspot promoter mutations in the TERT gene have represented the first recurrent oncogenic gain in MPM [31,32]. Moreover, pleura mesothelioma carrying the TERT promoter mutation with TP53 and NF2 mutations has demonstrated a shorter overall survival [33,34].

In a cohort of 29 patients with diagnoses of epithelioid MPM, we described 36 somatic mutations and 5 single-nucleotide polymorphisms. We did not find any mutations usually reported in MPM, such as *BAP1*, *CDKN2A* SNVs, etc.

The most pathologically relevant mutations in our series were identified in the *EGFR, KRAS, TP53* and *KDR* (VEGFR2) genes.

The *TP53* gene, located in chromosome 17 (p13.1), encodes for a tumor suppressor protein that acts as a transcriptional factor to induce cell cycle arrest, apoptosis, DNA repair, or changes in metabolism.

According to The Cancer Genome Atlas (TCGA), 16% of all MPM show a mutation in the *TP53* gene.

We detected six somatic mutations of *TP53*, all located in the typical hotspot of this onco-suppressor gene and corresponding to the DNA-binding domain. The highest allelic frequency (VAF: 32.6%) was found in the p.R175H variant, which has been demonstrated to confer oncogenic potential for the p53 protein, underlying a metastatic and chemo-resistant phenotype. This mutation consists of a substitution of arginine with histidine, causing a reduction of 100 times in the affinity for zinc and consequently leading to a structural remodeling of the protein. Na et al. demonstrated, in BRCA1 deficiency cell lines with concomitant TP53^R175H^, that the use of zinc metallochaperones (ZMCs) increased the zinc’s concentration and allowed for the correct folding of the mutant TP53 protein and its reactivation [35]. *TP53*-mutated MPM in our series represented 13.7% of the cases, although in the literature, it is more frequently reported in sarcomatoid and biphasic subtypes. Moreover, many studies have demonstrated its key role in determining an aggressive mesothelioma phenotype [31,36], mostly when associated with other genetic alterations probably related to a genomic instability [20,37].

In our cohort of epithelioid MPM, Kaplan–Meier analysis did not reveal any significant correlation between *TP53* mutations and OVS.

Recently, Sementino et al. [36] demonstrated in a mouse model that the combined inactivation of *PTEN* and *TP53* is able to drive the acquisition of an aggressive MPN phenotype through consistent activation of the AKT-mTOR signaling pathway. 

One of our patients carried concurrent somatic mutations in *PTEN* (p.Q245*), *AKT* (p.P51L) and *TP53* (p.R175H), demonstrating an overall survival of 32.2 months, in line with the median OS of epithelioid MPM. However, this represents only a single case, and more studies in human models are needed.

The *EGFR* (epidermal growth factor receptor) gene encodes for a transmembrane receptor tyrosine kinase belonging to a cell surface receptor super-family (RTK) that can be activated by several different ligands such as EGF (epidermal growth factor), TGFA (transforming growth factor-Alpha) and HBEGF (heparin-binding EGF-like growth factor) [32,38].

Ligand-dependent *EGFR* activation transduces multiple signaling pathways, including the RAS/MAPK pathway, the PI3K/AKT pathway, and the phospholipase C (PLC)/protein kinase C (PKC) signaling cascade, leading to cell proliferation, angiogenesis and inhibition of apoptosis [39].

It is well known that the EGFR gene is often mutated in numerous carcinoma, such as breast cancer, lung cancer and colon–rectal cancer.

In malignant mesothelioma, overexpression of the *EGFR* gene, which is detected by immunohistochemistry or FISH, is frequent, accounting for 44–90% of cases, and is probably caused by reactive oxygen species (ROS) present in the tumor microenvironment. EGFR mutations are infrequent, and in more than 75% of cases, they are rare mutations [29,37,40,41,42,43].

In 17.2% of the patients (5/29), we detected eight somatic variants of *EGFR*: two of them were located in exon 18 (p.G719S and p.A702D), one variant in exon 21 (p.E866K), and three mutations in exon 19 (p.T751I, p.S752Y and p.V774M). 

The SNVs occurring in codons 774, 719 and 751 were already described in other neoplasms from different sites. The variant in codon 702 has only been found in esophageal carcinoma [44].

We have also shown two *EGFR* mutations not yet described in MM, which are considered functionally pathogenetic, in both exon 18 (p.P694T) and exon 19 (p.S752C). Previously, the authors of [45] reported a pathogenetic *EGFR* mutation in exon 19 (p.E746_A750del), suggesting the potential role of EGFR tyrosine kinase inhibitors (EGFR TKIs) as target therapy in selected cases of MPM.

*KRAS* single-nucleotide variations in mesothelioma samples have been described for the first time in a study by Mezzapelle et al. [40], in which a correlation with OVS was not found. However, it was demonstrated that mutations in *KRAS* were not so rare, as reported in previous studies. 

Later, other MPM research studies based on comprehensive genomic analyses failed to find *KRAS* alterations due to the low frequency of copies of mutated alleles that require more sensitive approaches.

Recently, Trassl et al. [46] highlighted that although *KRAS* mutations are less commonly reported in MPM, *KRAS* pathway alterations are very frequent. Additionally, a study by Marazioti et al. [47] demonstrated, in a mouse model, that KRAS point mutations in epitheliod MPM, with or without TP53 alteration, are potentially able to drive MPM development and a more aggressive disease with frequent mesothelial pleural effusion. In this study, we reported five different somatic mutations in *KRAS* that occurred in 13.7% (4/29) of the patients. Notably, all the variants were characterized by a relatively small, below 5%, variant allele frequency (VAF), as usually reported in the literature, except in one case (*KRAS*:p.G13A) where the VAF was above 30%. Kaplan–Meier analysis revealed a negative impact of *KRAS* mutations on patient survival, confirming the data of previous studies, most of which were carried out on cell lines or murine models.

In detail, wild-type *KRAS* patients demonstrated a longer median overall survival (32.6 months; range: 26.7–38.5) when compared with patients harboring a *KRAS* mutation (median: 7.6 months, range: 4.6–10.4, HR (95%CI): 5.1 (1.5–17.9)).

Finally, another interest finding was the association between the single-nucleotide polymorphism *KDR* (VEGFR2) p.V297I (rs2305948) and a longer survival in MPM patients. Five of the patients showed this variant, and the statistical analysis indicated a relevant increase in median overall survival.

In the past few years, it has been shown that in both cases of pleuritis and pleural mesothelioma, an increase in serum level of vascular endothelial growth factor (VEGFR) occurs, particularly in mesothelioma [48,49]. Catalano et al. [50] demonstrated an inverse correlation between the serum level of VEGF and survival, suggesting that the VEGF signaling pathway can contribute to cancer progression.

The SNP p.V297I of *KDR* is caused by a missense point mutation in codon 297 located in exon 7, leading to the substitution of valine with isoleucine; this alteration produces a lower binding affinity between the receptor VEGFR2 (KDR) and its specific ligand VEGF, resulting in a softened function. In the literature, the role of this SNP is not yet defined, with a series of conflicting hypotheses. A few studies have described this substitution in *KDR* as an indicator of bad prognosis and lower overall survival [51,52,53], while the paper by Tinhofer in 2016 supports the hypothesis that it is a good prognostic factor in squamous cell carcinoma of the head and neck [54].

## 5. Conclusions

In this study, we found two mutations in exons 18 and 19 of the *EGFR* gene not previously described in the literature, both identified as potentially pathogenetic by different types of prediction software. In addition, we identified three mutations, p.V774M, p.T751I and p.S752Y, in exon 19 of *EGFR* not previously described in MPM.

Although a limitation of our study was the small number of cases studied and the lack of patient treatment data, we reported a possible correlation between *KRAS* mutations and shortened overall survival of patients when compared with wild-type cases. Previously, other studies conducted on cell lines [46] or on murine models [54] underlined the importance of the KRAS mutation in the pathogenesis and the progression of MPM. There are also extensive genomic and/or transcriptomic descriptive analyses of tissues from patients with MPM, showing among others the presence of a KRAS mutation. However, to the best our knowledge, the correlation between KRAS alteration and overall survival in primary neoplastic tissue samples is still lacking.

Moreover, another result that needs to be confirmed in large-scale studies is that ≥3 mutations had a negative influence on survival, compared with the patients carrying only one or two somatic variants.

Finally, at least in our cohort of patients, the presence of SNP p.V297I of the *KDR* gene seems to confer an advantage for overall survival.

Our study has weaknesses. First, it is retrospective in nature, and while eight patients were still alive at the time of the database lock, they all died or were lost by follow-up at the time of writing of this manuscript. Thus, information regarding therapy was available for only a few patients, which was uninformative as to the impact of treatment on survival. Second, the number of cases was relatively small and included the sole epitheliod MPM subtype. Third, pathological grading of MPM as well as the type of specimen procedures (biopsy or pleurectomy) showed no significant correlation with patient survival, but this may be related to the small amount of patients in our study.

Despite these limitations, our findings are in line with previous studies [45,46,47], pointing to an underestimated importance of the RAS pathway in pleura malignant mesothelioma, as suggested by a significantly worse prognosis for patients carrying *KRAS* mutations. This study adds some new findings in the molecular profile and prognosis of MPM patients, which remain to be fully validated in broader studies and in the different MPM histotypes.

## Figures and Tables

**Table 1:** Cohort’s clinical–pathological characteristics.

Patients. ID	Sex	Age	Alive at the Time of the Study	OS (Month)	Type of Specimen	Pathological Stage (pT)	Grading (Sec. WHO, 2021)
1	Male	73	No	10	Pleurectomy	pT1	2
2	Male	81	No	28.9	Biopsy	pT1	1
3	Male	67	No	7.6	Biopsy	pT1	1
4	Female	72	Yes	76.2	Pleurectomy	pT1	1
5	Male	68	No	5.8	Pleurectomy	pT1	2
6	Male	64	Yes	50.9	Biopsy	pT1	2
7	Male	73	No	27.2	Biopsy	pT1	1
8	Male	72	No	23.4	Biopsy	pT1	2
9	Male	63	No	37.6	Biopsy	pT1	1
10	Male	70	Yes	41.5	Pleurectomy	pT1	1
11	Female	73	No	9.7	Biopsy	pT1	3
12	Male	69	No	17.5	Biopsy	pT1	1
13	Male	70	No	32.2	Biopsy	pT1	1
14	Male	63	No	30.7	Pleurectomy	pT1	1
15	Female	65	Yes	29.4	Biopsy	pT1	2
16	Male	76	No	28.6	Biopsy	pT1	1
17	Male	67	No	27.3	Biopsy	pT1	2
18	Male	74	Yes	248.8	Biopsy	pT1	1
19	Male	70	No	25.7	Biopsy	pT1	1
20	Male	70	No	25.5	Biopsy	pT1	1
21	Male	83	No	14.3	Biopsy	pT1	2
22	Male	82	Yes	6.9	Biopsy	pT1	3
23	Male	84	No	32.6	Biopsy	pT1	1
24	Male	71	No	6.6	Biopsy	pT1	1
25	Male	69	Yes	8	Pleurectomy	pT1	2
26	Female	71	Yes	30.2	Biopsy	pT1	1
27	Male	67	No	1.1	Biopsy	pT1	3
28	Male	69	No	3.2	Biopsy	pT1	1
29	Male	80	No	10.2	Biopsy	pT1	2

**Table 2:** List of all the pathogenetic mutations found.

Gene	Exon	cHGVS	pHGVS	COSMIC_ID	VAF	Functions
KRAS	2	c.38G>C	p.G13A	COSM533	33.5	missense
	2	c.50G>A	p.S17N	COSM51382	6.36	missense
	3	c.169G>A	p.D57N	COSM166779	4.6	missense
	3	c.143G>A	p.G48E		4.2	missense
	3	c.142G>A	p.G48R		3.1	missense
EGFR	19	c.2320G>A	p.V774M	COSM13006	13.5	missense
	18	c.2155G>A	p.G719S	COSM6252	4.4	missense
	18	c.2105C>A	p.A702D	COSM3266631	5.5	missense
	19	c.2252C>T	p.T751I	COSM13185	11.8	missense
	21	c.2596G>A	p.E866K	COSM13198	9.5	missense
	19	c.2255C>G	p.S752C		7.7	missense
	19	c.2255C>A	p.S752Y	COSM13186	5.1	missense
	18	c.2080C>T	p.P694T	COSM13179	5.6	missense
TP53	5	c.524G>A	p.R175H	COSM10648	32.6	missense
	5	c.454C>T	p.P152S	COSM43582	9.6	missense
	6	c.637C>T	p.R213*	COSM99618	5.7	nonsense
	6	c.574C>T	p.Q192*	COSM10733	5.5	nonsense
	6	c.644G>A	p.S215N	COSM744739	4.3	missense
	6	c.672 + 1G>A	p.?	COSM6906	4.7	stop-loss
MET	14	c.3268G>A	p.G1090S		5.2	missense
	3	c.1113C>G	p.N371K		40.2	missense
CTNNB1	4	c.100G>C	p.G34R	COSM5685	4.9	missense
FGFR3	10	c.1138G>C	p.G380R	COSM9276948	5.8	missense
	15	c.1921G>A	p.D641N	COSM6953958	6.2	missense
BRAF	11	c.1390G>A	p.G464R	COSM1111	8.5	missense
STK11	4	c.182G>A	p.G61D	COSM6191357	3.8	missense
TSC2	23	c.2714G>A	p.R905Q	COSM4736821	7.9	missense
FLT3	20	c.2464G>T	p.G822W	COSM3885171	5.1	missense
AKT1	1	c.152C>T	p.P51L	COSM4468165	13.2	missense
HNF1A	3	c.599G>A	p.R200Q	COSM3457098	14.5	missense
	3	c.607C>T	p.R203C	COSM24915	5.9	missense
PTEN	6	c.733C>T	p.Q245*	COSM5159	6	nonsense
RB1	20	c.2048T>C	p.L683P		4.7	missense
CDH1	7	c.1081G>A	p.A361T		5.2	missense
MAP2K1	7	c.709G>T	p.G237W		8.17	missense
KIT	17	c.2473G>A	p.V825I	COSM19110	12.5	missense

## Data Availability

Data are contained within the article.

## References

[1] Travis W.D., Brambilla E., Nicholson A.G., Yatabe Y., Austin J.H.M., Beasley M.B., Chirieac L.R., Dacic S., Duhig E., Flieder D.B. (2015). The 2015 World Health Organization Classification of Lung Tumors: Impact of Genetic, Clinical and Radiologic Advances Since the 2004 Classification. J. Thorac. Oncol..

[2] Paajanen J., Laaksonen S., Ilonen I., Vehmas T., Mäyränpää M.I., Sutinen E., Kettunen E., Salo J.A., Räsänen J., Wolff H. (2020). Clinical Features in Patients with Malignant Pleural Mesothelioma With 5-Year Survival and Evaluation of Original Diagnoses. Clin. Lung Cancer.

[3] Furuya S., Chimed-Ochir O., Takahashi K., David A., Takala J. (2018). Global Asbestos Disaster. Int. J. Environ. Res. Public Health.

[4] International Agency for Research on Cancer (1977). IARC Monographs on the Evaluation of the Carcinogenic Risk of Chemicals to Man: Asbestos.

[5] Sauter J.L., Dacic S., Galateau-Salle F., Attanoos R.L., Butnor K.J., Churg A., Husain A.N., Kadota K., Khoor A., Nicholson A.G. (2021). The 2021 WHO classification of tumors of the pleura: Advances since the 2015 classification. J. Thorac. Oncol..

[6] van Meerbeeck J.P., Gaafar R., Manegold C., Van Klaveren R.J., Van Marck E.A., Vincent M., Legrand C., Bottomley A., Debruyne C., Giaccone G. (2005). Randomized phase III study of cisplatin with or without raltitrexed in patients with malignant pleural mesothelioma: An intergroup study of the European Organisation for Research and Treatment of Cancer Lung Cancer Group and the National Cancer Institute of Canada. J. Clin. Oncol..

[7] Verma V., Ahern C.A., Berlind C.G., Lindsay W.D., Shabason J., Sharma S., Culligan M.J., Grover S., Friedberg J.S., Simone C.B. (2018). Survival by Histologic Subtype of Malignant Pleural Mesothelioma and the Impact of Surgical Resection on Overall Survival. Clin. Lung Cancer.

[8] Sinn K., Mosleh B., Hoda M.A. (2021). Malignant pleural mesothelioma: Recent developments. Curr. Opin. Oncol..

[9] Janes S.M., Alrifai D., Fennell D.A. (2021). Perspectives on the Treatment of Malignant Pleural Mesothelioma. N. Engl. J. Med..

[10] Baas P., Scherpereel A., Nowak A.K., Fujimoto N., Peters S., Tsao A.S., Mansfield A.S., Popat S., Jahan T., Antonia S. (2021). First-line nivolumab plus ipilimumab in unresectable malignant pleural mesothelioma (CheckMate 743): A multicentre, randomised, open-label, phase 3 trial. Lancet.

[11] Mielgo-Rubio X., Cardeña Gutiérrez A., Sotelo Peña V., Sánchez Becerra M.V., González López A.M., Rosero A., Trujillo-Reyes J.C., Couñago F. (2022). Tsunami of immunotherapy reaches mesothelioma. World J. Clin. Oncol..

[12] Fennell D.A., Dulloo S., Harber J. (2022). Immunotherapy approaches for malignant pleural mesothelioma. Nat. Rev. Clin. Oncol..

[13] Tranchant R., Montagne F., Jaurand M.C., Jean D. (2018). Molecular heterogeneity of malignant pleural mesotheliomas. Bull. Cancer.

[14] Panou V., Roe O.D. (2020). Inherited Genetic Mutations and Polymorphisms in Malignant Mesothelioma: A Comprehensive Review. Int. J. Mol. Sci..

[15] Nymark P., Lindholm P.M., Korpela M.V., Lahti L., Ruosaari S., Kaski S., Hollmen J., Anttila S., Kinnula V.L., Knuutila S. (2007). Gene expression profiles in asbestos-exposed epithelial and mesothelial lung cell lines. BMC Genom..

[16] Wadowski B., De Rienzo A., Bueno R. (2020). The Molecular Basis of Malignant Pleural Mesothelioma. Thorac. Surg. Clin..

[17] Kang H.C., Kim H.K., Lee S., Mendez P., Kim J.W., Woodard G., Yoon J.H., Jen K.Y., Fang L.T., Jones K. (2016). Whole exome and targeted deep sequencing identify genome-wide allelic loss and frequent SETDB1 mutations in malignant pleural mesotheliomas. Oncotarget.

[18] Guo G., Chmielecki J., Goparaju C., Heguy A., Dolgalev I., Carbone M., Seepo S., Meyerson M., Pass H.I. (2015). Whole-exome sequencing reveals frequent genetic alterations in BAP1, NF2, CDKN2A, and CUL1 in malignant pleural mesothelioma. Cancer Res..

[19] Sato T., Sekido Y. (2018). NF2/Merlin Inactivation and Potential Therapeutic Targets in Mesothelioma. Int. J. Mol. Sci..

[20] Hmeljak J., Sanchez-Vega F., Hoadley K.A., Shih J., Stewart C., Heiman D., Tarpey P., Danilova L., Drill E., Gibb E.A. (2018). Integrative Molecular Characterization of Malignant Pleural Mesothelioma. Cancer Discov..

[21] Iacono M.L., Monica V., Righi L., Grosso F., Libener R., Vatrano S., Bironzo P., Novello S., Musmeci L., Volante M. (2015). Targeted next-generation sequencing of cancer genes in advanced stage malignant pleural mesothelioma: A retrospective study. J. Thorac. Oncol..

[22] Harrison P.T., Vyse S., Huang P.H. (2020). Rare epidermal growth factor receptor (EGFR) mutations in non small cell lung cancer. Semin. Cancer Biol..

[23] Barta J.A., Pauley K., Kossenkov A.V., McMahon S.B. (2020). The lung-enriched p53 mutants V157F and R158L/P regulate a gain of function transcriptome in lung cancer. Carcinogenesis.

[24] de Assis L.V., Isoldi M.C. (2014). The function, mechanisms, and role of the genes PTEN and TP53 and the effects of asbestos in the development of malignant mesothelioma: A review focused on the genes’ molecular mechanisms. Tumour. Biol..

[25] Sementino E., Menges C.W., Kadariya Y., Peri S., Xu J., Liu Z., Wilkes R.G., Cai K.Q., Rauscher F.J., Klein-Szanto A.J. (2018). Inactivation of Tp53 and Pten drives rapid development of pleural and peritoneal malignant mesotheliomas. J. Cell. Physiol..

[26] Tian K., Bakker E., Hussain M., Guazzelli A., Alhebshi H., Meysami P., Demonacos C., Schwartz J.M., Mutti L., Krstic-Demonacos M. (2018). p53 modeling as a route to mesothelioma patients stratification and novel therapeutic identification. J. Transl. Med..

[27] Hylebos M., Van Camp G., van Meerbeeck J.P., Op de Beeck K. (2016). The Genetic Landscape of Malignant Pleural Mesothelioma: Results from Massively Parallel Sequencing. J. Thorac. Oncol..

[28] Forde P.M., Anagnostou V., Sun Z., Dahlberg S.E., Kindler H.L., Niknafs N., Purcell T., Santana-Davila R., Dudek A.Z., Borghaei H. (2021). Durvalumab with platinum-pemetrexed for unresectable pleural mesothelioma: Survival, genomic and immunologic analyses from the phase 2 PrE0505 trial. Nat. Med..

[29] Hiltbrunner S., Fleischmann Z., Sokol E.S., Zoche M., Felley-Bosco E., Curioni-Fontecedro A. (2022). Genomic landscape of pleural and peritoneal mesothelioma tumours. Br. J. Cancer..

[30] Pagano M., Ceresoli L., Zucali P., Pasello G., Garassino M., Grosso F., Tiseo M., Parra H.S., Zanelli F., Cappuzzo F. (2020). Mutational Profile of Malignant Pleural Mesothelioma (MPM) in the Phase II RAMES Study. Cancers.

[31] Chia P.L., Scott A.M., John T. (2019). Epidermal growth factor receptor (EGFR)-targeted therapies in mesothelioma. Expert Opin. Drug Deliv..

[32] Agama N., Yasuda Y., Ozasa H. (2017). Malignant Pleural Mesothelioma Harboring Both G719C and S768I Mutations of EGFR Successfully Treated with Afatinib. J. Thorac. Oncol..

[33] Tallet A., Nault J.-C., Renier A., Hysi I., Galateau-Sallé F., Cazes A., Copin M.-C., Hofman P., Andujar P., Le Pimpec-Barthes F. (2014). Overexpression and promoter mutation of the TERT gene in malignant pleural mesothelioma. Oncogene.

[34] Cakiroglu E., Senturk S. (2020). Genomics and Functional Genomics of Malignant Pleural Mesothelioma. Int. J. Mol. Sci..

[35] Na B., Yu X., Withers T., Gilleran J., Yao M., Foo T.K., Chen C., Moore D., Lin Y., Kimball S.D. (2019). Therapeutic targeting of BRCA1 and TP53 mutant breast cancer through mutant p53 reactivation. NPJ. Breast Cancer.

[36] Bueno R., Stawiski E.W., Goldstein L.D., Durinck S., De Rienzo A., Modrusan Z., Gnad F., Nguyen T.T., Jaiswal B.S., Chirieac L.R. (2016). Comprehensive genomic analysis of malignant pleural mesothelioma identifies recurrent mutations, gene fusions and splicing alterations. Nat. Genet..

[37] Destro A., Ceresoli G.L., Falleni M., Zucali P.A., Morenghi E., Bianchi P., Pellegrini C., Cordani N., Vaira V., Alloisio M. (2006). EGFR overexpression in malignant pleural mesothelioma. An immunohistochemical and molecular study with clinico-pathological correlations. Lung Cancer.

[38] Sigismund S., Avanzato D., Lanzetti L. (2018). Emerging functions of the EGFR in cancer. Mol. Oncol..

[39] Mezzapelle R., Miglio U., Rena O., Paganotti A., Allegrini S., Antona J., Molinari F., Frattini M., Monga G., Alabiso O. (2013). Mutation analysis of the EGFR gene and downstream signalling pathway in histologic samples of malignant pleural mesothelioma. Br. J. Cancer.

[40] Govindan R., Kratzke R.A., Herndon J.E., Niehans G.A., Vollmer R., Watson D., Green M.R., Kindler H.L., Cancer and Leukemia Group B (2005). Gefitinib in patients with malignant mesothelioma: A phase II study by the Cancer and Leukemia Group B. Clin. Cancer Res..

[41] Schildgen V., Pabst O., Tillmann R.L., Lusebrink J., Schildgen O., Ludwig C., Brockmann M., Stoelben E. (2015). Low frequency of EGFR mutations in pleural mesothelioma patients, Cologne, Germany. Appl. Immunohistochem. Mol. Morphol..

[42] Shukuya T., Serizawa M., Watanabe M., Akamatsu H., Abe M., Imai H., Tokito T., Ono A., Taira T., Kenmotsu H. (2014). Identification of actionable mutations in malignant pleural mesothelioma. Lung Cancer.

[43] Liu Z., Chen Z., Wang J., Zhang M., Li Z., Wang S., Dong B., Zhang C., Gao J., Shen L. (2018). Mouse avatar models of esophageal squamous cell carcinoma proved the potential for EGFR-TKI afatinib and uncovered Src family kinases involved in acquired resistance. J. Hematol. Oncol..

[44] Kim J.E., Kim D., Hong Y.S., Kim K.P., Yoon Y.K., Lee D.H., Kim S.W., Chun S.M., Jang S.J., Kim T.W. (2018). Mutational Profiling of Malignant Mesothelioma Revealed Potential Therapeutic Targets in EGFR and NRAS. Transl. Oncol..

[45] Trassl L., Stathopoulos G.T. (2022). KRAS Pathway Alterations in Malignant Pleural Mesothelioma: An Underestimated Player. Cancers.

[46] Marazioti A., Krontira A.C., Behrend S.J., Giotopoulou G.A., Ntaliarda G., Blanquart C., Bayram H., Iliopoulou M., Vreka M., Trassl L. (2022). KRAS signaling in malignant pleural mesothelioma. EMBO Mol. Med..

[47] Duzkopru Y., Oruc Z., Kaplan M.A., Ulku R., Tanrikulu C., Esmer D., Birak A., Kucukoner M., Urakci Z., Isikdogan A. (2017). The importance of serum and pleural fluid level of vascular endothelial growth factor (VEGF) and VEGF fluid/serum ratio in the differential diagnosis of malignant mesothelioma-related pleural effusion. Contemp. Oncol..

[48] Hirayama N., Tabata C., Tabata R., Maeda R., Yasumitsu A., Yamada S., Kuribayashi K., Fukuoka K., Nakano T. (2011). Pleural effusion VEGF levels as a prognostic factor of malignant pleural mesothelioma. Respir. Med..

[49] Catalano A., Lazzarini R., Di Nuzzo S., Orciari S., Procopio A. (2009). The plexin-A1 receptor activates vascular endothelial growth factor-receptor 2 and nuclear factor-kappaB to mediate survival and anchorage-independent growth of malignant mesothelioma cells. Cancer Res..

[50] Geng N., Su J., Liu Z., Ding C., Xie S., Hu W. (2021). The Influence of KDR Genetic Variation on the Efficacy and Safety of Patients with Advanced NSCLC Receiving First-Line Bevacizumab Plus Chemotherapy Regimen. Technol Cancer Res. Treat.

[51] Bai M., Li Z.G., Ba Y. (2021). Influence of KDR Genetic Variation on the Efficacy and Safety of Patients with Chemotherapy Refractory Metastatic CRC Who Received Apatinib Treatment. Int. J. Gen. Med..

[52] Zheng Y.B., Huang J.W., Zhan M.X., Zhao W., Liu B., He X., Li Y., Hu B.S., Lu L.G. (2014). Genetic variants in the KDR gene is associated with the prognosis of transarterial chemoembolization treated hepatocellular carcinoma. Tumour. Biol..

[53] Tinhofer I., Stenzinger A., Eder T., Konschak R., Niehr F., Endris V., Distel L., Hautmann M.G., Mandic R., Stromberger C. (2016). Targeted next-generation sequencing identifies molecular subgroups in squamous cell carcinoma of the head and neck with distinct outcome after concurrent chemoradiation. Ann. Oncol..

[54] Patel M.R., Jacobson B.A., De A., Frizelle S.P., Janne P., Thumma S.C., Whitson B.A., Farassati F., Kratzke R.A. (2007). Ras pathway activation in malignant mesothelioma. J. Thorac. Oncol..

